# Supplementary material for: Analysis of chi angle distributions in free amino acids via multiplet fitting of proton scalar couplings
Source: Magn Reson (Gott). 2024 Aug 19;5(2):103–20. doi: 10.5194/mr-5-103-2024 (PMC11570886; doi:10.5194/mr-5-103-2024)
Supplement: The supplement related to this article is available online at: https://doi.org/10.5194/mr-5-103-2024-supplement. [file mr-5-103-2024-supplement.zip › Syed2024/data/fit1d_gissmo.tar.gz!/fit1d/tyr/500/bmse000051_simulation_1_nmredata.zip!/bmse000051_simulation_1_nmredata/1H/pdata/991/index.html_C=S_O=D]

Index of /ftp/pub/bmrb/metabolomics/standards/L\_tyrosine/nmr/bmse000051/1H/pdata/991


# Index of /ftp/pub/bmrb/metabolomics/standards/L\_tyrosine/nmr/bmse000051/1H/pdata/991

|  | Name | Last modified | Size | Description |
| --- | --- | --- | --- | --- |
| --- | | | | |
|  | Parent Directory |  | - |  |
|  | ct1t2.txt | 30-Sep-2011 09:43 | 2.3K |  |
|  | proc | 30-Sep-2011 09:43 | 1.8K |  |
|  | procs | 30-Sep-2011 09:43 | 1.8K |  |
|  | t1t2.dx | 30-Sep-2011 09:43 | 1.5K |  |
|  | t1peaks | 30-Sep-2011 09:43 | 1.4K |  |
|  | t1ints | 30-Sep-2011 09:43 | 1.4K |  |
|  | t1par | 30-Sep-2011 09:43 | 1.0K |  |
|  | t1peaks.txt | 30-Sep-2011 09:43 | 833 |  |
|  | t1ints.txt | 30-Sep-2011 09:43 | 833 |  |
|  | outd | 30-Sep-2011 09:43 | 520 |  |
|  | t1ascii | 30-Sep-2011 09:43 | 393 |  |
|  | t1\_disp | 30-Sep-2011 09:43 | 256 |  |
|  | dt1t2.txt | 30-Sep-2011 09:43 | 118 |  |
|  | pt1t2.par | 30-Sep-2011 09:43 | 90 |  |
|  | t1elim | 30-Sep-2011 09:43 | 60 |  |
| --- | | | | |

Apache/2.2.3 (CentOS) Server at www.bmrb.wisc.edu Port 80
